# Imaging of retroperitoneal haemorrhage revealing median arcuate ligament syndrome

**DOI:** 10.4102/sajr.v25i1.1993

**Published:** 2021-01-15

**Authors:** Younes El Hassani, Meriem Haloua, Badreeddine Alami, Meryem Boubbou, Mustapha Maaroufi, Moulay Youssef Alaoui Lamrani

**Affiliations:** 1 Department of Radiology, Faculty of Medicine and Pharmacy, Sidi Mohammed Ben Abdellah University, Hassan II University Hospital, Fez, Morocco

**Keywords:** median arcuate ligament syndrome, haematoma, pancreaticoduodenal aneurysm, retroperitoneal haemorrhage, epigastric pain

## Abstract

Coeliac artery compression stenosis caused by the median arcuate ligament can lead to aneurysm formation in the pancreatico-duodenal arteries that can eventually result in a spontaneous rupture leading to retroperitoneal haemorrhage. In this case series, we describe the cases of three patients, all presenting with sudden epigastric pain, diagnosed as spontaneous haematoma, complicating a median arcuate ligament syndrome.

## Introduction

Median arcuate ligament syndrome (MALS) is a rare condition related to symptoms that occur as a result of extraluminal compression of the coeliac artery (CA) by the median arcuate ligament (MAL).^1^

In MALS, blood flow to the splanchnic organs is diverted to the superior mesenteric artery (SMA) and then to the pancreatico-duodenal arcades because of decreased flow in the coeliac trunk. The pancreatico-duodenal arteries (PDAs) cannot sustain this high flow, leading to true-aneurysm formation, which can eventually result in spontaneous rupture.^2,3^

In this case series, we describe the case presentations of three patients, all presenting with sudden epigastric pain, diagnosed as spontaneous haematoma complicating MALS.

## Patient presentations

### Case 1

A 65-year-old man presented to the emergency department for atraumatic acute abdominal pain and vomiting. He was a chronic tobacco user with no medical or surgical history or any regular medication.

Upon arrival, he presented with normal vital parameters. Abdominal examination revealed epigastric tenderness. He was treated initially with intravenous paracetamol before performing an ultrasound (US) which demonstrated the presence of a heterogenous mass in the epigastric region and the presence of a hyperechoic, well-organised heteregenous mass within the right iliac fossa, with no Doppler signal.

**FIGURE 1:**
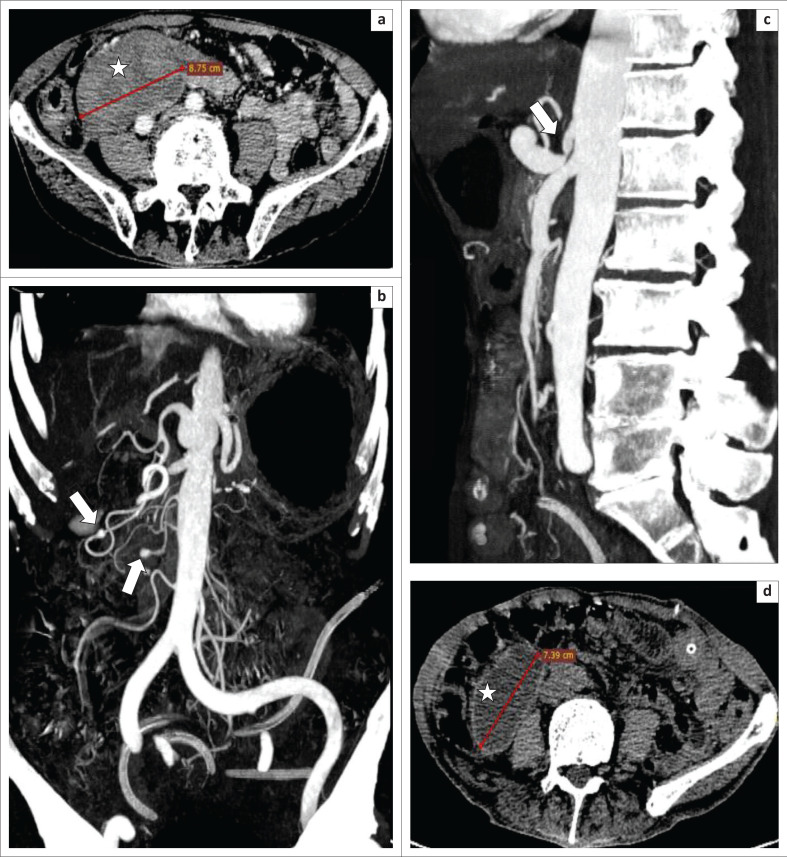
A 65-year-old man with atraumatic acute abdominal pain and vomiting: Arterial phase contrast enhanced computed tomography of the abdomen in the axial (a), coronal (b) and sagittal planes (c) show a well-orgnised mesenteric haematoma (asterix, a) of approximately 9 cm. Two small aneurysms were identified (arrows, b) originating from the upper and lower pancreatico-duodenal arteries. There was significant coeliac artery compression by the median arcuate ligament (arrow, c) resulting in post-stenotic fusiform dilatation of the celiac artery. Non-enhanced computed tomography in the axial plan (d), performed 2 months later, shows homogeous liquifecation of the haematoma with a well-distinguished regular and thin wall.

Thereafter, abdominal computed tomography (CT) with contrast was performed and demonstrated a well-organised and hyperdense mesenteric haematoma measuring with mean density of 57 Hounsfield unit (HU) approximately 9 cm × 5 cm × 4 cm. Two small aneurysms were identified originating from the upper and lower PDAs. There was marked CA compression by the MAL resulting in post-stenotic fusiform dilatation of the CA (Figure 1).

### Case 2

A 64-year-old woman with persistent abdominal pain for two weeks was admitted to our emergency department. The patient complained of diffuse abdominal pain and haematemesis; digital rectal exam indicated melena. Clinical abdominal examination revealed a peri-umbilical palpable mass with localised tenderness. Eso-gastro-duodenal endoscopy (EGDE) displayed external compression of the second-part duodenum without any mucosal abnormality. Biochemistry demonstrated regenerative microcytic and hypochromic anaemia.

An abdominal US was performed, highlighting the presence of free hyperechoic fluid in the pouch of Douglas. No mass was identified.

**FIGURE 2:**
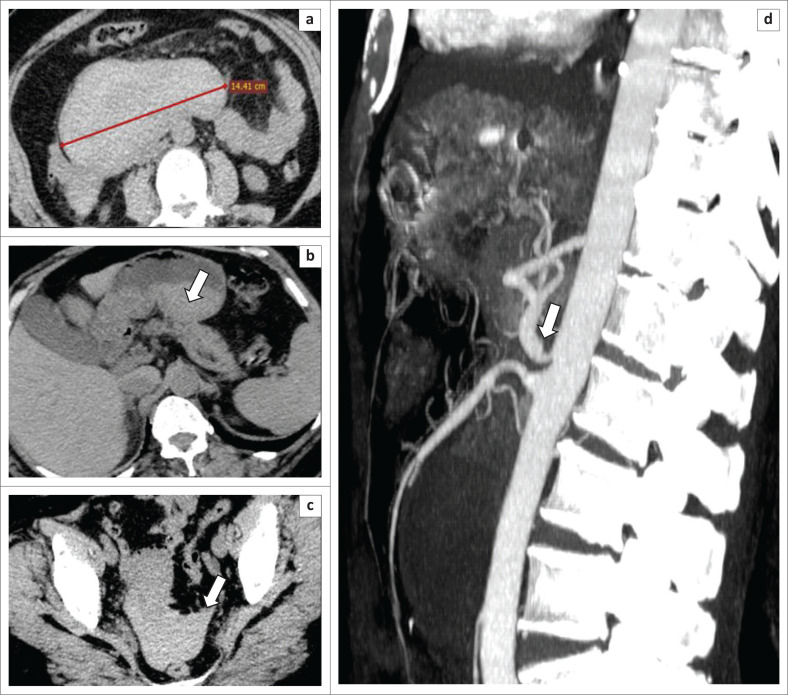
A 64-year-old woman with persistent diffuse abdominal pain, admitted for haematemesis and melena. Axial non-enhanced computed tomography demonstrates a homogeneous hyperdense haematoma of approximately 14 cm (a), haemorrhage within the stomach (arrow, b) and haemoperitoneum (arrow, c). Arterial phase sagittal reconstruction (d) demonstrates moderate coeliac artery compression by the median arcuate ligament with no visible pancreatico-duodenal artery aneurysms.

Subsequent abdominal CT demonstrated a homogenous hyperdense haematoma (43 HU) of approximately 14 cm × 10 cm × 8 cm with haemorrhage within the stomach (average density of 62 UH). There was moderate CA compression by the MAL with no visible PDA aneurysm (Figure 2).

### Case 3

A 44-year-old woman was admitted for acute abdominal and back pain, as well as haematemesis and melena. Her previous medical and surgical history was unremarkable and she was not on any regular medication.

Upon arrival, the patient had a tachycardia and was tachypnoeic. She was normotensive, had normal oxygen saturation (98%) and was afebrile. Abdominal examination elicited diffuse tenderness.

Eso-gastro-duodenal endoscopy did not show any abnormalities. Her blood tests were normal apart from a modestly raised C-reactive protein (20 mg/dL) and mildly decreased haemoglobin levels (9 g/dL).

**FIGURE 3:**
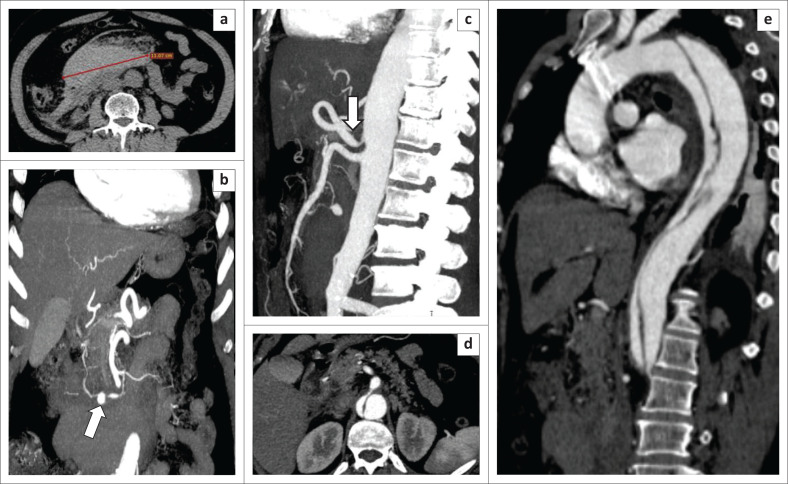
A 44-year-old woman admitted with haematemesis and melena. Non-enhanced computed tomography in the axial plane (a) showing a homogeneous hyperdense haematoma of approximately 11 cm. Arterial phase in coronal (b), sagittal (c), axial (d) and oblique plan (e) revealed a small aneurysm (arrow, b) originating from the inferior pancreatic-duodenal artery (IPDA) (arrow, b); Moderate coeliac artery compression by the median arcuate ligament (arrow, c) is identified. There is a Standford B aortic dissection (e) where the coeliac artery originantes from the false lumen (d).

Abdominal CT revealed a retroperitoneal haematoma with rupture of a small aneurysm originating from the inferior pancreatic-duodenal artery (IPDA), secondary to stenosis of the CA. Additionally, there was also a Standford B dissection of the aorta. It is postulated that the above scenario was caused by the decreased blood flow in the CA compressed by the intimal flap (causing dynamic ischaemia), which then resulted in further decreased flow in the coeliac trunk, increased blood flow in SMA branches and finally rupture of the pre-existing aneurysm (Figure 3).

## Discussion

Median arcuate ligament syndrome or CA compression syndrome (CACS), also called Dunbar syndrome, is a rare condition in which the MAL causes compression of the CA. It is characterised clinically by weight loss, postprandial epigastric pain, nausea and vomiting.^4^ The mean age at diagnosis is 28 years with a 71% female preponderance.^5^ However, only 10% – 24% of patients with extrinsic compression of the CA by the MAL will have the syndrome.^6^

The relation between the anatomic anomaly and clinical symptoms was first described by Harjola in 1963, in a case study, and then by Dunbar in 1965 in a larger case series of 15 subjects.^7^

### Pathophysiology

The pathophysiology is clearly established. In MALS, blood flow to the splanchnic organs is diverted to the SMA and then to the PDA because of decreased flow in the CA. The PDAs cannot sustain this high flow, causing high-flow true aneurysms, which can eventually result in spontaneous rupture.^2^ Aneurysm regression or stability is reported after CA reconstruction with stent placement, MAL incision or bypass placement.

There are other causes of PDA aneurysms other than MALS such as atherosclerosis, pancreatitis, mycotic and bacterial infection, trauma or fibromuscular hyperplasia.^2,8^

### Imaging findings

The radiological diagnosis of MALS is firstly based on detecting MAL thickening equal to or greater than 4 mm,^9^ but the definitive finding for the diagnosis is focal narrowing of the proximal CA with a characteristic ‘hooked appearance’, best seen on a sagittal image.^10^ This sign helps differentiating MALS from other causes of CA stenosis, such as atherosclerosis. Associated findings include post-stenotic dilatation or collateral vessel formation from the SMA branches.^6^

Computed tomography and magnetic resonance imaging (MRI) should ideally be performed in the end-inspiratory and expiratory phases. True and permanent compressions persist or increase during end-expiration, whereas transient physiological compression is seen only during end-inspiration.^6^

Percutaneous angiography is the reference standard for the diagnosis of MAL syndrome and shows findings similar to CT such as superior indentation, hooking and post-stenotic dilatation of the CA. Furthermore, angiography can assess the stenosis in both end-inspiration and expiration. Retrograde filling of the CA from the PDA can also be assessed.^6^

Doppler US can demonstrate peak systolic velocities greater than 200 cm/s correlating to a degree stenosis of at least 70% with a sensitivity of 75% and specificity of 89%.^7,11^

Median arcuate ligament syndrome may cause true post-stenotic aneurysms, which can be located anywhere in the distal arteries, but appear to predominantly involve the PDA and dorsal pancreatic artery (DPA) – the two main collateral networks between the CA and the SMA.^12,13^

True aneurysms of the PDA are rare, accounting for only 2% of all visceral aneurysms.^13^ Almost half of all PDA aneurysms are associated with CA stenosis.^14^ Compression by the MAL has been shown to be responsible for stenosis in about 10% – 30% of cases.^14^ They can also be induced by periarterial inflammation, usually associated with pancreatitis, trauma, infection or atherosclerosis.^13^

More than 60% of patients with PDA aneurysms initially present with a rupture.^13^ Rupture risk is not related to the aneurysm size, suggesting that all aneurysms should be treated, regardless of their size.^15^ In the literature, ruptured aneurysm diameters ranged between 0.7 cm and 2 cm. On the other hand, unruptured aneurysm sizes were between 0.7 cm and 6 cm.^16^ The rupture risk of PDA aneurysms is about 65%. Among the case histories of 88 patients with PDA aneurysm published in the literature, 53 patients had rupture and 26 of them unfortunately died.^15^

In the third reported case, there was a Standford B aortic dissection where the CA originated from the false lumen. We postulate that this caused dynamic ischaemia and reduction of blood flow through the CA. The intimal flap reduces the CA ostium size and potentiates blood reduction caused by the abnormal MAL, resulting in further increased blood flow in the SMA branches, and finally, rupture of the small IPDA aneurysm. To the best of our knowledge, no such case was reported elsewhere.

The issues of treatment options regarding PDA aneurysms are still controversial. Recently, endovascular treatment has become popular but open surgical repair is also an option for young patients with no malignancy, for whom long-term survival is expected.^15^

## Conclusion

Rupture of PDA aneurysms caused by MALS should always be considered in the differential diagnosis of acute abdominal pain and retroperitoneal haemorrhage, as the condition requires specific management. Embolisation has been proven to be safe with a high success rate, with surgery reserved for unsuccessful embolisation.
